# Target discovery and drug design in the era of artificial intelligence

**DOI:** 10.1007/s00044-026-03562-1

**Published:** 2026-04-23

**Authors:** Payton Fleming, Andrey A. Ivanov

**Affiliations:** 1 Department of Pharmacology and Chemical Biology, Emory University School of Medicine, Emory University, Atlanta, GA 30322, USA; 2 Emory Chemical Biology Discovery Center, Emory University School of Medicine, Emory University, Atlanta, GA 30322, USA; 3 Winship Cancer Institute, Emory University, Atlanta, GA 30322, USA

**Keywords:** Artificial intelligence, machine learning, target discovery, molecular design, ADMET prediction

## Abstract

The identification of therapeutically actionable targets and the design of effective small-molecule modulators remain central challenges in drug discovery, particularly for complex diseases driven by dynamic, interconnected molecular networks. Recent advances in artificial intelligence (AI) and machine learning are reshaping this landscape by enabling integrative analysis of large-scale biological data, systematic navigation of chemical space, and data-driven optimization of molecular properties. In this review, we outline how AI transforms small-molecule drug discovery by linking target discovery, tractability assessment, and chemical design within integrated, experiment-informed workflows. We highlight AI-enabled strategies for target discovery that integrate network biology, multimodal data fusion, and perturbation-aware modeling. These approaches identify context-specific, functionally actionable targets, including non-enzymatic proteins, protein-protein interactions, and other historically challenging classes. We then focus on advances most relevant to medicinal chemistry, including structure and complex prediction, docking and affinity modeling, ligand-based learning, generative molecular design, ADMET prediction, and synthetic feasibility assessment. Finally, we discuss current limitations related to data quality, generalizability, interpretability, and experimental translation. We emphasize the critical future role of integrated, context-aware AI workflows that connect target discovery with chemical design and experimental feedback. Together, these advances position AI as a robust framework for accelerating the discovery of therapeutically relevant targets and small-molecule modulators.

## Graphical Abstract

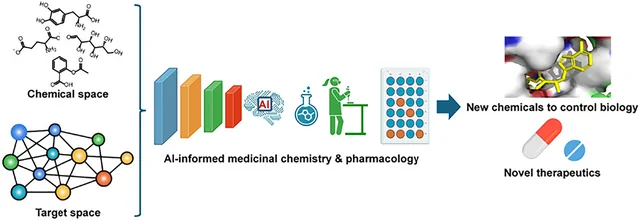

## Introduction

The identification of therapeutically actionable molecular targets and the design of small-molecule modulators remain foundational to modern drug discovery. Advances in molecular biology, genomics, structural biology, and medicinal chemistry have created unprecedented opportunities to explore disease biology with greater precision and depth. However, the inherent complexity of human biology, driven by dynamic, multiscale molecular interactions, continues to challenge traditional approaches to target selection and optimization.

Complex diseases such as cancer, neurodegeneration, metabolic disorders, and chronic inflammatory conditions arise from the dysregulation of interconnected molecular networks that adapt dynamically to genetic, environmental, and therapeutic perturbations [1, 2]. While conventional experimental approaches have provided invaluable mechanistic insights, they often fall short in capturing the emergent behaviors of these systems. Addressing this complexity requires integrative frameworks that synthesize data across molecular, cellular, and systems levels to derive actionable biological insight.

Artificial intelligence (AI) and machine learning (ML) have rapidly emerged as transformative tools capable of meeting this challenge [3, 4]. By integrating high-dimensional, heterogeneous datasets, AI enables the identification of non-obvious target-disease associations, uncovers hidden structure-function relationships, and supports hypothesis generation [5]. Rather than replacing experimental discovery, AI complements it, serving as a bridge that connects molecular data with biological understanding and therapeutic decision-making.

**Fig. 1:**
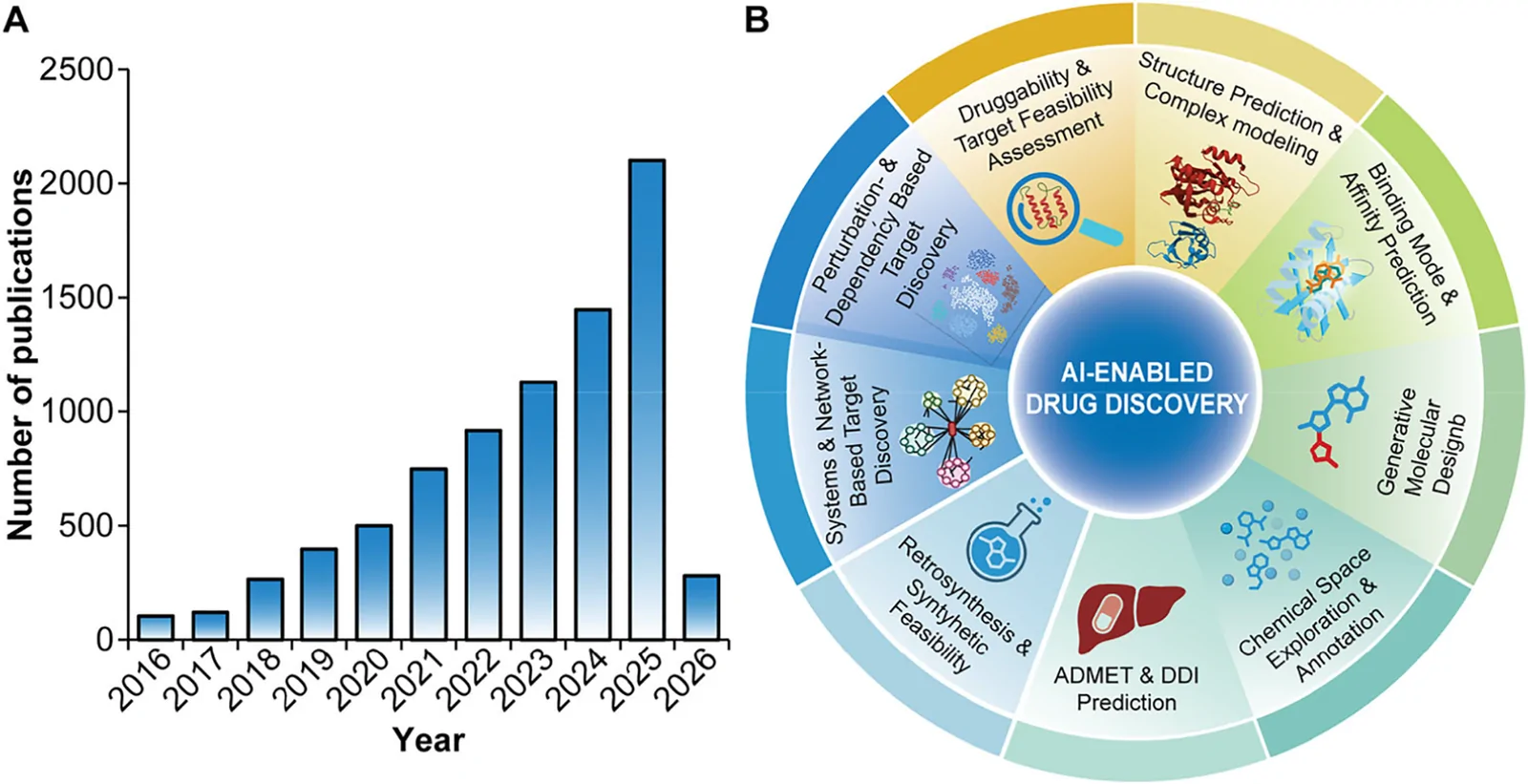
Rapid growth and application landscape of artificial intelligence in medicinal chemistry and drug discovery. **A** The bar graph shows a constantly growing number of PubMed-indexed publications referring to AI/ML methodologies in the context of medicinal chemistry, drug discovery, molecular design, QSAR, or cheminformatics. **B** Representative areas of target and drug discovery facilitated by AI approaches

Recent advances in deep learning architectures, graph-based representation learning, and generative chemistry have accelerated the use of AI across all stages of early target and drug discovery (Fig. 1). These methods now inform target identification and validation, virtual screening, de novo molecule generation, multi-parameter optimization, and developability assessment. For example, AI-driven molecular design platforms have integrated structure-based and ligand-based methods to efficiently explore ultra-large chemical spaces while improving the prediction of drug-target interactions [3, 6].

The translational impact of these approaches is increasingly evident, with several AI-designed small molecules advancing into clinical evaluation, demonstrating the feasibility of AI-augmented drug discovery pipelines [7, 8]. These developments underscore AI’s potential to enhance the precision, speed, and efficiency of discovery efforts, offering a framework to better align molecular insights with therapeutic outcomes.

In this review, we provide a comprehensive overview of AI-enabled strategies for target discovery and small-molecule design. We emphasize systems- and network-level perspectives, highlight advances in computational-experimental integration, and discuss how AI is transforming medicinal chemistry. Finally, we outline key challenges, including data quality, interpretability, and integration into discovery workflows, and identify future directions that will be essential to realize AI’s full potential in translating data into effective therapeutics.

## AI for target discovery

Target discovery has traditionally relied on linkage analysis, differential expression, and biochemical annotation to associate genes or proteins with disease phenotypes, yielding many successful therapeutics and foundational mechanistic insight. Increasingly, these approaches are complemented by AI-enabled strategies that integrate large-scale molecular data, functional perturbation evidence, and systems-level context to prioritize actionable disease drivers rather than isolated molecular signals. A broad range of computational resources, datasets, and AI methodologies, including network- and systems-based frameworks, perturbation-aware models, dependency mapping resources, and multimodal learning approaches, now support target discovery across biological scales, enabling more context-aware and functionally grounded prioritization. Representative tools and methods are summarized in Table 1. Below, we highlight key AI-driven target discovery approaches, beginning with network- and systems-based methods and extending to perturbation-aware and context-specific frameworks.

### Network- and systems-based approaches

Target discovery has traditionally focused on identifying genes or proteins associated with disease phenotypes through linkage analysis, differential expression, or biochemical annotation. These approaches have yielded numerous successful therapeutics and continue to offer critical mechanistic insights. Increasingly, however, they are complemented by systems-level network biology frameworks that capture the collective behavior of interacting molecular components. In this view, disease is considered an emergent property of complex, adaptive networks, characterized by robustness, modular organization, and distributed regulation rather than isolated molecular defects [9].

Cellular processes can be represented as interconnected graphs of proteins, genes, and metabolites, encompassing protein-protein interaction (PPI) networks, signaling circuits, and metabolic pathways. Within these networks, topology encodes key biological constraints and organizational principles [10]. Empirical studies have shown that highly connected “hub” proteins often perform essential cellular roles, whereas intermediate “bottleneck” nodes regulate information flow and system resilience [11]. Together, these insights have inspired target discovery strategies that incorporate network topology, modularity, and pathway connectivity into target prioritization.

To enable scalable network-based discovery, platforms such as NDEx (Network Data Exchange), integrate curated, experimental, and AI-inferred networks [12]. Such infrastructure enables AI models to directly integrate and compare network representations across studies [13]. In parallel, the development of disease-focused interactome resources, such as the OncoPPi network of cancer-associated PPIs, has illustrated how cancer-associated proteins form highly structured subnetworks centered around critical hubs, including MYC, AKT1, or STK11, revealing new routes for therapeutic interrogation of non-enzymatic targets [14, 15]. Network-based approaches have also been applied to neurodegenerative disease, where causal drivers are subtle and distributed across molecular systems. Integration of protein co-expression and transcriptomic networks in Alzheimer’s disease revealed early-emerging, glial-centered protein modules that were more strongly associated with cognitive decline and pathology than RNA-based signals alone, illustrating how protein-centric network organization can expose actionable disease mechanisms in complex disorders [16].

The emergence of AI-enabled network analysis has expanded this framework by introducing algorithms that can learn and propagate biological signals across large-scale interaction maps. *Random-walk* and *diffusion-based* approaches, for instance, disseminate information from known disease genes through the interactome to reveal functionally related neighborhoods and potential mediators [17] (Box 1). These approaches are particularly effective for multifactorial diseases, such as cancer or neurodegeneration, where integrating subtle, polygenic effects across pathways can highlight convergent biological mechanisms and therapeutic opportunities. In parallel, machine learning platforms such as AVERON now enable the quantification of mutation-induced neomorphic PPIs (neoPPIs) and their clinical impact across tumor types, uncovering actionable oncogenic programs and novel dependencies [18].

The network medicine paradigm builds upon these developments by showing that disease-associated genes tend to cluster within disease modules d - connected subnetworks representing shared biological functions [19]. Mapping these modules provides an integrative framework to uncover candidate targets, biomarkers, and cross-disease relationships. This modular view aligns naturally with the biological reality that multiple signaling routes can converge on common cellular states, such as inflammation, metabolic adaptation, or stress response, and supports the prioritization of targets with the greatest capacity to modulate key network hubs.

Recent advances in graph representation learning and graph neural networks (GNNs) have further elevated the potential of network-based discovery. These models integrate network topology with node-specific attributes, such as gene expression, mutation profiles, and perturbation responses, into unified, context-aware embeddings that enable node classification, link prediction, and module detection [20, 21]. In n particular, GNNs and related AI architectures can identify novel dependencies in cancer cell lines, reveal latent regulatory structures in complex tissues, and predict synthetic lethal pairs for combination therapy design.

Recent AI-driven advances have expanded network-based target discovery beyond static, curated interactomes by inferring PPI structure directly from sequence and contextual molecular data. Sequence-based approaches such as diffPALM use masked protein language modeling on paired protein sequences to learn latent interaction signatures encoded in amino acid context, enabling prediction of interacting protein pairs without reliance on existing interaction maps [22]. Complementarily, InteracTor applies large protein language models to embed individual protein sequences into a shared latent space and score interaction likelihoods at the proteome scale, supporting systematic expansion of interaction networks beyond experimental and literature bias [23]. Addressing the context dependence of protein interactions, SPIDER integrates general interactome structure with condition-specific molecular features using graph attention networks to infer cell-type-specific PPI networks, improving identification of disease-relevant modules and targets in tissue- and state-specific settings [24]. Together, these methods extend network-based target discovery by integrating sequence and contextual information beyond static topology.

Complementing graph neural networks, Discriminative Network Embedding (DNE) methods have shown that rich functional information can be extracted directly from protein-protein interaction topology, even in the absence of extensive node-level annotations [25]. DNE frameworks learn both local and global interaction patterns through contrastive self-supervision, enabling accurate detection of functional modules and interaction relationships across large-scale human interactomes. These topology-driven representations outperform classical random-walk and autoencoder-based embeddings in module discovery and link prediction, highlighting their ability to capture biologically meaningful organization without reliance on expression or mutation features. Such approaches are particularly valuable for target discovery in data-sparse settings, reinforcing the utility of AI-enabled network modeling for identifying non-enzymatic and regulatory targets embedded within disease-associated interaction modules.

Network- and systems-based methods are particularly powerful for illuminating non-enzymatic and regulatory targets, such as scaffolding proteins, transcriptional adaptors, or signaling regulators, that influence cellular state through connectivity rather than catalytic activity. In oncology and genomics, for instance, large-scale loss-of-function studies have shown that network context helps explain why certain dependencies emerge in specific genetic or environmental backgrounds [17, 26]. For example, OncoPPi-derived mapping of cancer-associated protein-protein interactions revealed that the histone methyltransferase isoform NSD3S, which lacks catalytic activity, binds to MYC via a defined 15-amino-acid region to block its ubiquitin-mediated degradation by FBXW7, thereby extending MYC protein half-life and transcriptional output [27]. Complementary mechanistic work identified the dual-specificity kinase MKK3 as a non-canonical MYC interactor that stabilizes and transcriptionally activates MYC through a short MYC-binding motif, independent of its kinase function. This MKK3-MYC PPI emerges as an attractive and mechanistically distinct therapeutic target in cancer, as its pharmacological disruption by first-in-class inhibitors [28] suppresses tumor growth in vivo and restores sensitivity to EGFR inhibitors, including overcoming acquired resistance to osimertinib in EGFR-mutant lung cancer [29].

Incorporating dynamic and perturbation-resolved information further enhances network interpretability and translational potential. Differential network biology highlights that condition-specific interactions and temporal dynamics often reveal the most actionable mechanisms for intervention [30]. As single-cell, spatial, and multi-omics datasets become increasingly accessible, network approaches are being refined with cell-type and tissue-specific resolution, connecting molecular modules to physiological states and disease progression [31, 32].

Together, these innovations position network- and systems-based frameworks as central to the next generation of target discovery. By combining the conceptual strength of systems biology with the computational power of AI, researchers can now capture the complexity of disease networks with unprecedented depth, illuminating novel therapeutic targets and pathways poised for clinical translation.

### Multimodal integration approaches

#### Rationale for multimodal integration in target discovery

A defining advantage of artificial intelligence lies in its capacity to integrate heterogeneous data modalities into unified predictive frameworks that capture the complexity of human disease biology. Modern target discovery increasingly relies on the joint analysis of genomics, transcriptomics, proteomics, phosphoproteomics, epigenomics, metabolomics, chemical perturbation data, and clinical annotations [33]. Each modality provides a complementary and often orthogonal view of biological state, and no single data type is sufficient to identify causal disease drivers with high confidence, motivating integrative and multi-omics analysis strategies [34, 35].

Genomic data can reveal inherited risk variants and somatic alterations, yet such changes do not always translate into functional dependency or therapeutic vulnerability in disease-relevant contexts. Transcriptomic profiles capture dynamic gene expression programs but are frequently confounded by cell-state heterogeneity and technical noise. This limitation is amplified in bulk measurements and motivates the increasing reliance on single-cell and multimodal profiling [31, 36]. Proteomic and phosphoproteomic measurements provide closer proximity to functional signaling activity and pathway engagement, strengthening mechanistic inference beyond transcript abundance alone [37]. Epigenomic layers encode longer-term regulatory potential and can support interpretation of cell identity and state transitions in health and disease [38]. Metabolomic and lipidomic profiles reflect pathway-level outputs, while chemical perturbation data offer direct insight into functional dependencies [39–41]. Clinical phenotypes and outcomes, in turn, anchor molecular observations to disease relevance and therapeutic impact [42, 43]. AI-based multimodal integration seeks to unify these layers into coherent, biologically meaningful representations that can support robust target prioritization and drug discovery [44].

#### Representation learning for multimodal data fusion

Deep learning architectures have proven particularly effective for multimodal data fusion by modeling coordinated pathway activity and regulatory dependencies across complex biological datasets. Autoencoder-based representation learning has been used to denoise and compress high-dimensional biological datasets, including sparse single-cell measurements, while preserving underlying biological structure [45]. Variational autoencoders (VAEs) have similarly been applied to learn biologically meaningful representations from large transcriptomic compendia, enabling downstream inference and prediction tasks relevant to target discovery [46, 47]. For example, VEGA [47] introduced an interpretable variational autoencoder architecture whose latent variables are explicitly constrained to represent biological pathways and gene regulatory modules, enabling direct inference of pathway and transcription factor activity from single-cell RNA-seq data. This AI-driven latent representation revealed cell-type-specific activation of interferon signaling and tryptophan catabolism pathways in stimulated immune cells, identified drug-specific pathway responses across cancer cell lines, and recovered known master regulators such as STAT3 and OLIG2 in glioblastoma, insights that were not detectable using conventional differential expression or gene set enrichment analyses alone. Furthermore, the development of multimodal integration methods developed for single-cell data has provided a conceptual blueprint for AI-driven fusion across modalities by explicitly aligning datasets, learning shared representations of cell state, and preserving modality-specific signals [48–50]. These models can learn both modality-specific features and shared latent representations, allowing them to identify relationships that were not apparent in any single dataset.

#### Functional target prioritization beyond differential expression

A key strength of multimodal AI approaches is their ability to prioritize targets based on functional importance rather than the magnitude of change in any single modality. For example, a protein may exhibit only modest transcriptional regulation yet occupy a critical position within a regulatory network or display heightened sensitivity in perturbational datasets. By integrating expression programs with functional perturbation evidence, AI models can elevate such proteins as high-confidence therapeutic targets despite weak signals in traditional differential analyses.

Emerging multimodal AI frameworks further demonstrate how integrating perturbational, molecular, and phenotypic signals refines functional target prioritization beyond static association metrics. The Cell Maps for Artificial Intelligence (CM4AI) project provides a large-scale, systematically generated resource that combines genetic perturbation screens, quantitative proteomics, protein-protein interaction mapping, and cellular phenotyping across diverse biological contexts [51]. CM4AI-derived perturbation-aware deep learning approaches jointly model molecular interventions alongside transcriptional and proteomic responses to infer functionally effective intervention points within cellular networks. Rather than relying solely on expression magnitude, these methods prioritize coordinated pathway responses and state transitions as indicators of therapeutic relevance, recognizing that even modest molecular changes can have significant functional consequences when viewed in a network context.

For medicinal chemistry, these frameworks provide a rationale for prioritizing targets where modest expression signals mask strong functional leverage, focusing resources on mechanisms that are actionable even when transcriptional changes are subtle. For example, PDGrapher [52] identifies sets of candidate targets whose modulation shifts diseased transcriptional states toward treated phenotypes. It further prioritizes network-effective targets, such as KDR (VEGFR2) in non-small-cell lung cancer, based on their ability to reverse disease expression programs rather than on their differential expression magnitude. Geneformer [53] leverages large-scale single-cell pretraining and in silico gene deletion to identify dosage-sensitive and disease-modifying regulators. For instance, in cardiomyopathy it recovered experimentally validated targets such as TEAD4, whose functional impact is not apparent from expression changes alone. In addition, PINNACLE [54] integrates single-cell transcriptomics with PPI networks to nominate cell type-specific therapeutic targets. Its application in inflammatory diseases, correctly prioritized established disease drivers such as TNF and IL6R, based on their contextual interaction topology and cellular specificity, even when transcriptional changes are modest.

Multimodal integration is particularly transformative in precision medicine contexts, where disease heterogeneity and context dependence are dominant features. In oncology, AI-driven integration of molecular profiles with drug-response data has enabled prediction of response mechanisms and rational prioritization of vulnerabilities and combination opportunities [55]. The DrugCell framework [55] integrates tumor genotypes with drug chemical structure in an interpretable deep neural network, enabling simultaneous prediction of drug response and identification of cellular subsystems mediating sensitivity and resistance. This approach revealed non-obvious pathway-level determinants of drug response, such as metabolic and signaling subsystems governing sensitivity to paclitaxel and etoposide, that were not recoverable from single-gene biomarkers or differential expression analyses. By ranking functionally important subsystems, the model directly nominated synergistic drug combinations, including PI3K or MAPK pathway inhibitors with chemotherapeutics, which were experimentally validated in vitro, by combinatorial CRISPR perturbations and patient-derived xenograft models [55].

Complementing these strategies, large-scale dependency resources, such as the Dependency Map (DepMap) Project [56], provide a functional layer for distinguishing actionable dependencies from genomic passengers. For example, a translational cancer dependency map (TCGA-DEPMAP) trained on CRISPR loss-of-function data from DepMap cell lines and projected onto patient tumors from The Cancer Genome Atlas identified lineage-specific oncogene dependencies, clinically actionable synthetic lethal interactions, and vulnerabilities predictive of therapeutic response and survival [57]. Notably, this framework uncovered previously underappreciated synthetic lethality, such as PAPSS1-PAPSS2 co-dependency driven by collateral deletion with PTEN, which was experimentally validated in vitro and in vivo and shown to correlate with adverse clinical outcomes.

Beyond cancer, multimodal AI approaches are increasingly applied to inflammatory and neurodegenerative diseases, where causal drivers are often subtle, distributed, and cell-type specific. Integration of single-cell transcriptomics with other modalities enables the identification of disease-associated cell states and regulatory programs that are not recoverable from bulk measurements [58, 59]. AI-based multimodal models can link these molecular signatures to disease stage, progression, and clinical outcomes, facilitating the discovery of targets that modulate pathological processes without disrupting essential homeostatic functions. In Alzheimer’s disease, for example, single-cell transcriptomic analyses have identified disease-associated microglial states regulated by TREM2 that coordinate phagocytosis, lipid metabolism, synaptic remodeling, and inflammatory signaling [60]. AI-assisted frameworks integrating single-cell omics, neuroimaging, and clinical data further illustrate how distributed, cell-state-specific molecular programs can be translated into actionable targets for the intervention of neurodegenerative and inflammatory diseases.

#### Chemical perturbations and closed-loop multimodal discovery

Chemical perturbation datasets, such as the Connectivity Map (CMap) project and its expanded L1000 platform [61], provide a critical functional layer for multimodal integration, enabling systematic mapping between genes, pathways, and small molecules. Recent advances in artificial intelligence have leveraged these large-scale perturbational resources to model the complex relationships among chemical structure, transcriptional response, and cellular phenotype. Deep learning models trained on L1000 perturbation data, such as DrugReflector [62], demonstrate that transcriptomic signatures can predict compounds that modulate disease-relevant states, including known pathway inhibitors and novel inducers of hematopoietic differentiation. Likewise, the CSG^2^A transfer learning framework integrates L1000, GDSC, and TCGA data to model condition-specific gene-drug interactions, revealing context-dependent vulnerabilities and compensatory pathways that support target de-risking [63].

The multimodal integration principles are increasingly applied within closed-loop discovery platforms that couple biological data fusion with molecular design and experimental feedback. For example, systems such as PandaOmics [64] integrate multi-omics, clinical annotations, and knowledge graphs to prioritize disease-relevant targets, while Chemistry42 [65] connects generative molecular design with active learning and experimental validation. In these frameworks, multimodal representations guide both target selection and compound optimization, enabling iterative refinement of hypotheses as new experimental data is generated. Such platforms exemplify how multimodal AI transitions from analytical integration to executable discovery workflows.

These studies highlight that integrating AI-based perturbation modeling with molecular and clinical data not only enables systematic mapping across biological scales but also provides a robust framework for predictive and mechanistically grounded drug discovery. Multimodal AI approaches represent a critical evolution in target discovery, enabling a shift from single-modality analyses toward integrated, systems-level prioritization. As data quality improves and integrative models mature, these approaches are poised to play a central role in identifying robust, context-aware therapeutic targets across diverse disease areas.

### Druggability prediction

Biological relevance alone is insufficient for successful drug development. Targets must also be amenable to pharmacological modulation. This distinction was formalized by the concept of the druggable genome [66], which highlighted that only a subset of proteins, particularly enzymes, receptors, and ion channels, has historically yielded successful drugs. Early druggability assessments, therefore, favored proteins with deep, well-defined binding pockets, reflecting historical success rather than intrinsic biological importance. In contrast, modern computational and AI-based approaches treat druggability as a continuous, site- and context-dependent property that can be inferred from sequence- and structure-derived features. This perspective has motivated machine-learning frameworks that support both target-level prediction from protein sequence and site-level ligandability assessment from structure. For example, DrugProtAI applies supervised machine-learning models to sequence-derived amino acid composition and physicochemical descriptors to enable rapid, proteome-wide classification of proteins as druggable or non-druggable without requiring structural information [67].

Related sequence-based approaches further emphasize feature integration and model robustness. DrugnomeAI extends target-level druggability prediction by combining multiple sequence-derived encodings with machine-learning classifiers to improve discrimination between druggable and non-druggable proteins across diverse functional classes [68]. Similarly, XGB-DrugPred employs extreme gradient boosting trained on optimized feature sets, including group dipeptide composition and reduced amino acid alphabets, to enhance predictive accuracy and reduce redundancy in high-dimensional sequence feature spaces [69]. These methods share a common objective of excluding globally intractable targets rather than identifying specific binding sites.

Ensemble learning strategies have proven particularly effective for improving the robustness of sequence-based druggability prediction. A Bagging-SVM approach systematically combines multiple support vector machine classifiers trained on bootstrapped subsets of sequence-derived features, improving generalization and robustness compared to single-model predictors [70]. Building on this concept, SPIDER implements a stacked ensemble-learning architecture that integrates heterogeneous base classifiers and feature representations into a meta-predictor, achieving improved performance and interpretability for druggable protein identification [71].

These ensemble methods are optimized for early-stage target prioritization but do not assess site-specific ligandability. In contrast, structure-based approaches resolve tractability at the level of individual binding sites, reflecting the local nature of ligand binding. P2Rank represents a machine-learning pocket detection tool that predicts ligandability by scoring local chemical neighborhoods on the solvent-accessible surface of proteins using random forest models trained on physicochemical descriptors [72]. Designed for speed and automation, P2Rank is well-suited for large-scale pocket annotation and integration into structure-based drug discovery pipelines.

Deep learning methods further enhance site-level druggability prediction by learning spatial representations directly from three-dimensional protein structures. For instance, DeepSite introduced a 3D convolutional neural network that encodes proteins as voxelized grids with atom-type channels, enabling direct learning of complex spatial patterns associated with ligand-binding pockets [73]. This approach demonstrated that ligandability is encoded in higher-order structural features that are not captured by classical geometric rules. DeepPocket also builds on this paradigm by combining initial geometric pocket detection with deep learning-based rescoring, improving the identification and ranking of true ligand-binding sites among numerous candidate cavities [74].

Beyond static pocket detection, some methods address binding-site quality and selectivity in highly dynamic targets, like RNA. DRLiPS (Druggable RNA-Ligand binding Pocket Selector) represents a structure-based machine-learning framework that predicts RNA binding-site druggability using RNA-specific structural descriptors and supervised learning trained on curated RNA-ligand complexes [75]. By distinguishing ligandable RNA pockets from non-functional cavities and demonstrating sensitivity to conformational variation and point mutations, DRLiPS complements general pocket detection tools by refining site-level prioritization for selective small-molecule binding.

Taken together, these approaches provide complementary strategies for druggability assessment. Sequence-based models operate at the whole-protein level to enable rapid, proteome-wide target triage, whereas structure-based and deep learning approaches resolve druggability at the level of individual binding sites, supporting rational prioritization for docking, fragment screening, and medicinal chemistry optimization. By integrating these complementary layers, modern AI-driven workflows move beyond historical druggability assumptions toward a multi-resolution, data-driven assessment of pharmacological feasibility.

### Overarching challenges in target discovery

Advances in network-based analysis, multimodal integration, and AI-driven druggability prediction have substantially expanded the scope of target discovery. However, their effective application is challenged by several factors:

#### Uneven distribution of biological knowledge and data

Biological interaction maps, functional annotations, and perturbation datasets remain unevenly represented across genes and disease contexts. This imbalance influences the resolution and confidence of AI-based inference despite advances in representation learning and network propagation. The impact of AI-driven target discovery therefore depends critically on the availability of high-quality experimental data to anchor computational predictions and enable iterative validation and refinement.

#### Context-dependent target relevance

Target relevance varies across cell types, disease stages, and microenvironments. Single-cell and spatial profiling have demonstrated that key disease programs may be restricted to specific cellular states that are obscured in bulk analyses. AI models must therefore preserve cellular resolution and explicitly account for biological heterogeneity.

#### Bridging disease association and therapeutic effect

Many AI approaches successfully identify disease-associated genes, pathways, or network modules. However, therapeutic relevance depends on whether modulating a candidate alters disease biology. Functional genomics resources provide insight into context-specific dependencies, yet genetic perturbations do not always recapitulate pharmacological modulation. Integrating perturbation data with structural and chemical evidence remains essential for evaluating therapeutic potential.

#### Alignment between biological relevance and chemical feasibility

Network and multimodal approaches prioritize targets based on system-level influence, whereas druggability prediction evaluates chemical feasibility at the protein or binding-site level. Integrating these complementary perspectives enables coherent and actionable target prioritization across diverse disease contexts.

Systematically addressing these factors will be critical for realizing the full potential of AI-driven target discovery across diverse disease contexts.

## AI for small-molecule design

The origins of artificial intelligence in chemical modeling mark a pivotal transition in the evolution of computational chemistry. In the early 1990s, neural networks were introduced as nonlinear alternatives to classical quantitative structure-activity relationship (QSAR) models, challenging the limitations of traditional linear regression and descriptor-based frameworks. Early investigations demonstrated that feed-forward neural networks could identify complex, multidimensional patterns linking molecular structure to biological activity, significantly outperforming conventional models [76]. Subsequent analyses comparing linear and nonlinear QSAR approaches highlighted both the potential and challenges of the early AI systems, which still remain the major bottlenecks for modern models, including sensitivity to overfitting, difficulties in interpretation, and dependence on data quality [77]. Despite these limitations, these pioneering studies represented an important conceptual shift, showing that molecular activity could be modeled through adaptive, data-driven learning rather than fixed statistical equations. These early studies laid the foundation for the modern era of AI in chemistry, setting the stage for today’s deep learning architectures that now drive predictive modeling and molecular design across drug discovery (Box 2). Fueled by advances in deep learning, the availability of large-scale molecular datasets, and unprecedented computational power, the field is now undergoing exponential growth, rapidly reshaping the landscape of modern molecular design and discovery.

### Modern chemical space

**Fig. 2:**
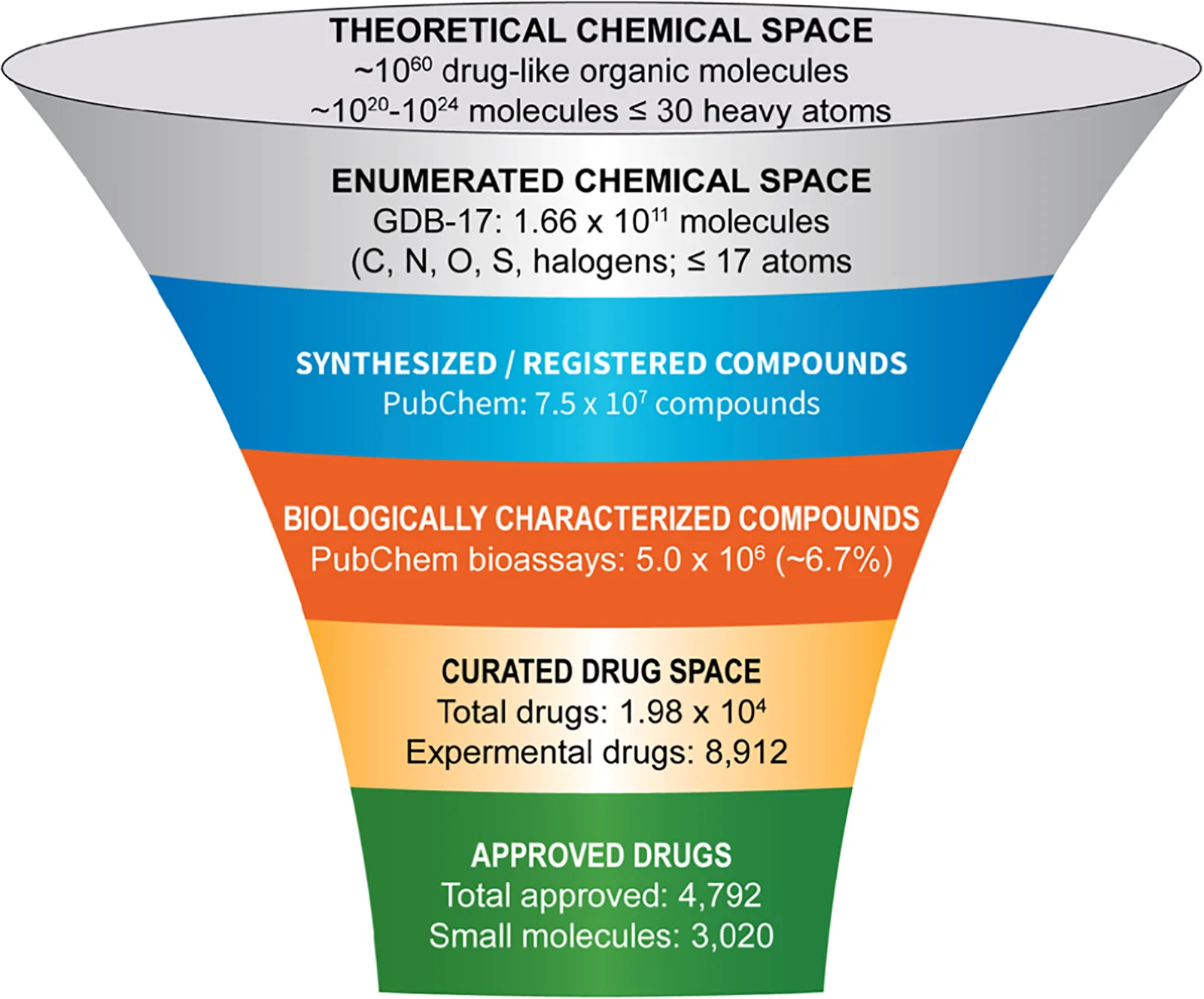
The modern chemical space. The funnel diagram illustrates the dramatic narrowing from the vast theoretical chemical space of drug-like molecules to the small number of approved drugs. While billions of compounds can be enumerated and millions have been synthesized and biologically tested, only a tiny fraction progress to curated drug space and eventual regulatory approval, highlighting the sparsity of validated therapeutics within overall chemical space

Chemical space defines the theoretical and practical limits within which small-molecule drug discovery operates. Estimates of its total size underscore its enormity. The number of chemically valid organic molecules compatible with basic valency and stability rules has been projected to exceed 10^60^ when constrained to traditional “drug-like” physicochemical ranges, and to approximately 10^20^–10^24^ for molecules containing up to ~ 30 heavy atoms (Fig. 2).

The Chemical Space Project [78] generated the Chemical Universe Databases (GDBs), most notably GDB-17, which contains 166.4 billion unique molecules composed of C, N, O, S, and halogens with up to 17 atoms, after filtering for chemical stability and synthetic plausibility. These results demonstrate both the depth of feasible chemistry and the extreme sparsity of experimentally explored regions.

Historically, navigation of chemical space has been guided by empirical frameworks such as “drug-likeness,” reflecting physicochemical properties common to approved oral drugs [79, 80]. These principles have provided highly informative and practical guidance for compound prioritization and remain central to medicinal chemistry practice. At the same time, their interpretation is increasingly recognized as context-dependent, particularly as discovery efforts expand to include non-canonical targets, allosteric sites, and protein-protein interfaces. Analyses of enumerated databases such as GDB-17 [78] showed that many chemically stable molecules fall outside classical drug-like definitions, motivating a shift toward data-driven, target-specific prioritization rather than universal property thresholds.

The chemical space of synthesized compounds represents the experimentally realized subset of the enumerated chemical space. Public repositories such as PubChem [81] and ChEMBL [82] compile tens of millions of structurally distinct molecules synthesized and registered over decades of medicinal chemistry and screening efforts. As of January 2026, PubChem lists 75,356,419 unique compounds. However, only 5,050,118 compounds are annotated with biological test results. Thus, fewer than 10% of registered molecules have been evaluated in biological assays. This limited biological coverage underscores the sparsity of functionally characterized chemical space and reveals significant opportunities to discover novel bioactive compounds within existing collections.

At the clinically validated end, the number of successful drugs is orders of magnitude smaller. According to DrugBank [83], the total number of curated drugs across all categories is 19,828, encompassing approved, experimental, nutraceutical, illicit, and withdrawn compounds. Of these, only 4791 drugs are currently approved for human use, including 3019 approved small-molecule drugs and 4350 biotech drugs (with overlap across regulatory classifications). The remaining entries include 8912 experimental drugs that have not reached approval, 914 withdrawn drugs, 135 nutraceuticals, and 205 illicit substances. This break-down highlights the substantial reduction from synthesized or tested compounds to clinically approved therapeutics, underscoring both the stringency of drug development and the narrow region of chemical space that has been successfully translated into medicines.

In addition to synthesized compounds, virtual chemical space comprises chemically plausible yet unsynthesized structures. Reaction-based enumeration and make-on-demand strategies have produced ultra-large libraries, reaching from billions to trillions of compounds, that extend far beyond experimentally realized chemistry. For instance, extensive make-on-demand libraries such as Enamine REAL and WuXi GalaXi have been constructed from validated reaction schemes using readily available building blocks, enabling the exploration of over 20 billion synthetically accessible molecules [84]. Large-scale virtual screening campaigns have similarly docked 14 million fragments and 235 million lead-like molecules, representing approximately 13 trillion virtual complexes, by combining reaction-derived catalogs with in silico enumeration methods [85]. More focused reaction-encoded virtual libraries have also been constructed around modular synthetic schemes. For example, the docking of 14.6 million synthetically tractable compounds derived from such a scheme enabled the discovery of potent ligands from underrepresented regions of chemical space [86].

Taken together, modern chemical space is best viewed as a continuum spanning millions of biologically tested compounds, tens of millions of synthesized molecules, tens of thousands of curated drug candidates, thousands of approved medicines, hundreds of billions of explicitly enumerated structures, and trillions of synthetically accessible virtual compounds. Empirical databases provide essential grounding and biological annotation, while enumerated and virtual spaces reveal the true depth of feasible chemistry. Advances in AI-driven molecular modeling and prioritization are enabling systematic exploration of chemical space, uncovering new classes of small molecules that modulate diverse protein classes beyond traditional targets, such as PPI-stabilizing molecular glues [87].

### Structure-based AI approaches

Structure-based drug design (SBDD) has been transformed by artificial intelligence (AI) methods that extend from protein structure prediction to complex modeling, pose generation, and binding affinity estimation. Rather than treating structural information as a scarce experimental resource, modern AI makes structure a computationally accessible layer that can be generated, stress-tested, and iterated early, often before any in-house structure is solved [88, 89]. This has shifted design logic from “find a structure, then design” to “design with predicted structures, then refine experimentally.” In practice, AI now acts as a front- end hypothesis engine, rapidly proposing plausible complex geometries, ranking ligands or pockets, and triaging chemical matter, followed by physics-based and experimental refinement.

#### AI-enabled structure and complex prediction

A persistent bottleneck in classical SBDD was the limited availability of high-resolution protein structures, particularly for membrane targets and dynamic complexes. Homology modeling enabled structure-based hypothesis generation well before experimental structures were available and, in some cases, reproduced core folds and binding-site layouts with excellent accuracy [90]. However, systematic retrospective analyses revealed that errors in loop architecture, side-chain placement, and pocket detail often limited its reliability for predictive drug design [91].

AlphaFold2 demonstrated that deep learning can overcome these limitations, achieving near-experimental accuracy for many protein structures directly from sequence [89]. Subsequent proteome-scale deployment extended reliable structural coverage to most human proteins, enabling early pocket analysis, construct design, and structure-guided hypothesis generation prior to the availability of experimental structures [92]. A second generation, AlphaFold3, extended this paradigm to biomolecular interactions, including protein-protein, protein-nucleic-acid, and protein-ligand complexes, through a diffusion-based architecture [88]. This advance is directly relevant to drug discovery, as many therapeutically actionable sites are complex-defined, emerging or reorganizing upon partner binding or cofactor association. Benchmarking efforts, including CASP evaluations [93], have delineated Alpha-Fold3’s current reliability across such mixed-component targets. However, persistent limitations have been revealed, most notably for long flexible loops and RNA geometries [94], where predictions remain uncertain and echo limitations previously observed in homology modeling.

#### AI-assisted docking and pose estimation

Binding-pose prediction remains central to structure-based drug design because it informs how chemical structure shapes target engagement and downstream pharmacology. Recent advances in deep learning have augmented this process in several complementary ways. Learned rescoring and refinement approaches, exemplified by GNINA [95, 96], integrate three-dimensional convolutional neural networks into classical docking pipelines to rescore poses and improve enrichment relative to empirical scoring functions. These methods yield substantial gains in large-scale virtual screening, where even modest improvements in ranking accuracy can translate into higher hit rates.

In parallel, machine learning-based docking approximation strategies such as Deep Docking [97] employ deep regression models trained on a subset of docked compounds to predict docking scores for the remainder of ultra-large libraries. This approach reduces the need for exhaustive docking by up to two orders of magnitude while preserving enrichment of top-ranked ligands [98]. More integrated frameworks, including AI-accelerated pipelines such as RosettaVS [6], combine learned scoring with structural sampling to jointly optimize binding poses and predicted affinities, improving both accuracy and throughput in prospective screening applications. In practice, these AI-assisted approaches are most effective when combined with physics-based sampling, where they prioritize plausible geometries, filter low-quality poses, and guide downstream molecular-dynamics or free-energy refinement rather than replacing them outright. Collectively, these strategies reduce the cost of hit discovery by enriching for experimentally tractable chemotypes earlier, allowing medicinal chemistry efforts to focus on compounds with a higher likelihood of experimental validation.

Recent advances have further extended AI-assisted docking beyond pose rescoring and ligand placement toward unified modeling of full protein-small-molecule assemblies. RoseTTAFold All-Atom (RFAA) introduces a three-track deep learning architecture that jointly represents proteins, nucleic acids, metals, covalent modifications, and small molecules at atomic resolution, enabling end-to-end prediction of protein-ligand complex structures directly from sequence and chemical graph inputs [99]. Unlike classical docking pipelines that assume a fixed receptor structure, RFAA simultaneously predicts protein backbone, side-chain conformations, and ligand poses, capturing induced-fit effects and multicomponent assemblies in a single forward pass. Benchmarking on blind protein-ligand modeling tasks demonstrates improved accuracy relative to conventional docking workflows when receptor structures are unknown or only partially resolved. These capabilities position all-atom deep learning models as a complementary layer to traditional docking, particularly for challenging targets lacking high-quality experimental structures or involving complex ligand chemistry.

#### AI-based affinity modeling

Where pose estimation answers *how* a ligand might bind, affinity prediction addresses *how strongly*. Deep learning has advanced this domain through both three-dimensional convolutional and graph-based architectures trained on structure-affinity datasets. KDEEP [100] and Pafnucy [101] introduced 3D CNNs to capture geometric interaction fields, while graph-based frameworks such as PotentialNet improved binding-affinity and physicochemical-property prediction across benchmark datasets [102]. These methods serve as effective triage tools for ranking analogs, prioritizing series, and identifying liabilities before expensive free-energy or experimental validation. The latest generation, exemplified by Boltz-2, seeks to unify structure prediction and affinity estimation in a single foundation model that bridges the gap between geometric accuracy and energetic fidelity [103]. Boltz-2 has also been applied at the proteome scale to model PPI networks, demonstrating how joint structure-affinity learning can support interface tractability analyses across the interactome [104].

Large-scale prospective evaluation of co-folding methods has provided a more realistic view of their strengths and limitations in structure-based ligand discovery. Using a benchmark of 557 previously unpublished ligand-protein crystal structures for the SARS-CoV-2 Mac1 macrodomain, recent work [105] showed that diffusion-based co-folding models, including AlphaFold3, Chai-1 [106], and Boltz-2, can reproduce a substantial fraction of ligand poses with near-atomic accuracy. In many cases, pose recovery exceeded that of classical docking. Importantly, prediction accuracy was largely independent of ligand similarity to the training set, indicating genuine generalization rather than memorization.

Despite these advances, limitations remain. The models struggled to capture larger ligand-induced conformational changes, particularly loop rearrangements. Their ability to distinguish true binders from false positives in large virtual screening hit lists was also target-dependent and often inferior to physics-based docking. Among co-folding methods, Boltz-2 showed the strongest correlation between predicted affinity and experimental potency. However, its role differs from that of docking. Boltz-2 does not replace large-scale physics-based screening. Instead, it provides a learned, structure-aware affinity estimate that is weakly correlated with docking scores. This complementarity supports hybrid workflows in which docking performs rapid triage across large libraries, while Boltz-2 is applied downstream to refine pose-affinity relationships within confirmed ligand series [105].

To address remaining limitations in pose control and site specificity, a conditional diffusion strategy has recently been introduced. This approach constrains receptor structure and guides ligand sampling using explicit pocket and hotspot information. Implemented in SiteAF3 [107], it improves complex structure prediction across protein-small-molecule, protein-peptide, and protein-nucleic-acid benchmarks. Gains were particularly evident for allosteric ligands, orphan targets lacking close structural homologs, and systems with low-confidence or flexible binding sites. SiteAF3 improves pose accuracy relative to unconstrained co-folding models while also reducing computational cost by narrowing the generative search space, highlighting the value of conditional diffusion for site-aware structure-based ligand discovery.

Together, AI-driven structure-based drug design is particularly powerful when structural and ligand data are limited, such as during early target validation or hit discovery, while also providing substantial value at later stages through structure-guided lead optimization and refinement. In these settings, predicted protein structures and complexes can reveal plausible binding pockets, accelerate compound triage, and improve enrichment in docking campaigns [6]. At the same time, predicted structures often capture dominant conformations rather than full bioactive ensembles, pose predictions may be overconfident, and affinity models remain sensitive to training-set biases. As a result, the most reliable workflows follow an AI-physics-experiment hierarchy, integrating rapid hypothesis generation with mechanistic refinement and experimental validation to support early-stage discovery.

### Modern AI approaches in ligand-based drug design

Ligand-based drug design (LBDD) exploits relationships between chemical structure and biological activity to predict molecular properties and guide compound design without requiring explicit knowledge of the target three-dimensional structure. Recent advances in artificial intelligence (AI) have substantially expanded the scope of LBDD, moving the field beyond classical descriptor-based quantitative structure-activity relationship (QSAR) models toward more flexible, data-driven frameworks [108]. These approaches (i) learn expressive molecular representations directly from chemical graphs or molecular language, (ii) scale naturally to multitask and multi-property objectives, and (iii) support prospective discovery workflows in which model predictions are experimentally tested.

Whereas classical QSAR approaches relied primarily on linear or low-capacity nonlinear models applied to fixed descriptor sets, modern ML-enabled QSAR frameworks learn task-specific molecular representations and support prospective prediction, prioritization, and decision-making in medicinal chemistry workflows [44, 109]. Deep learning architectures, including graph-based neural networks, have demonstrated improved performance when sufficient high-quality data are available, while ensemble and probabilistic methods such as random forests and Gaussian processes remain widely used owing to their robustness, interpretability, and principled uncertainty estimates in data-limited settings [110]. In practice, modern LBDD pipelines increasingly combine graph neural network (GNN) encoders with multitask and self-supervised learning, uncertainty estimation, and model interpretation frameworks, often integrated with chemical language or physicochemical features, enabling robust and data-efficient decision-making in exploratory discovery settings.

#### Learned molecular representations for ligand-based modeling

A defining shift in modern LBDD is the replacement of fixed fingerprints and hand-crafted descriptors with learned molecular representations that generalize across endpoints. Self-supervised pretraining on large, unlabeled chemical collections enables models to learn general structural and statistical patterns in molecules, which can then be adapted for property prediction using relatively small labeled datasets. Among these approaches, chemical language models have emerged as a powerful representation framework. For instance, MG-BERT, which adapts masked-token learning to SMILES strings, demonstrates consistent improvements across diverse molecular property prediction benchmarks following pretraining, underscoring the value of general-purpose chemical embeddings for QSAR-like applications [111]. Related approaches further refine this paradigm by incorporating chemically meaningful tokens into the language model itself. FG-BERT extends masked self-supervision to functional groups, enabling the model to learn representations aligned with chemically meaningful substructures commonly used in medicinal chemistry, while maintaining strong performance across physicochemical, ADMET, and phenotypic prediction benchmarks [112].

In parallel, supervised graph neural networks (GNNs) have become a dominant ligand-based prediction framework, learning message-passing directly over atom-bond graphs from labeled bioactivity data. Directed message-passing architectures achieve state-of-the-art performance across MoleculeNet benchmarks [113], including solubility and toxicity prediction, and substantially outperform traditional random forest models under scaffold-based splits, highlighting their ability to capture chemically meaningful features that generalize beyond close analogs [114]. Graph convolutional families such as PotentialNet have been developed to predict protein-ligand binding affinities for drug-like ligands and other key molecular properties with superior accuracy relative to conventional scoring functions [102]. Building on this, GNN-based models have also achieved unprecedented performance in predicting ADMET properties for collections of drug-relevant small molecules, improving lead triage and prioritization in medicinal chemistry workflows [115]. In related supervised settings, GNNs trained on drug response data have been used to predict cancer cell line sensitivity across hundreds of marketed anticancer agents, demonstrating the ability of graph models to learn structure-activity relationships relevant to real drugs [116].

Prospective validation has become a defining hallmark of modern AI-driven LBDD, reflecting the ability of data-driven models to prioritize chemically diverse compounds for experimental testing. Although these models are trained on compounds with known activity, their value lies in their capacity to generalize beyond narrow analog series and extrapolate across broader regions of chemical space. Rather than focusing primarily on manually selected compound subsets, models trained on historical or newly generated screening data are deployed to rank large and structurally diverse libraries for prospective evaluation. This shift is illustrated by deep learning-guided phenotypic screening for antibacterial activity, in which neural networks trained on growth inhibition data were used to prioritize chemically diverse compounds for testing. This strategy led to the identification of halicin, followed by extensive validation across bacterial species and in vivo infection models [117]. Although focused on anti-infectives, this work established a generalizable blueprint for phenotypic LBDD: learning structure-phenotype relationships from known activity data and extrapolating beyond the training distribution. This approach was sub-sequently extended to pathogen-specific discovery. Using a training set of approximately 7500 screened compounds, a deep learning model prioritized molecules active against Acinetobacter baumannii, culminating in the discovery of abaucin, with confirmed mechanism-of-action studies and efficacy in a mouse wound infection model [118]. Similarly, in the context of immune checkpoint modulation, a machine learning-based QSAR framework integrating molecular fingerprints with ensemble classifiers was used to prioritize PD-1/PD-L1 interface inhibitors from large chemical libraries. Subsequent docking, molecular dynamics simulations, and experimental validation led to the identification of a novel small-molecule scaffold capable of disrupting the PD-1/PD-L1 interaction [119] (Table 2).

As LBDD models increase in complexity, interpretability has evolved from a diagnostic add-on to a practical design tool. Explainable AI approaches aim not only to rationalize predictions but also to extract chemically actionable insights that inform lead optimization. In an explainable deep learning study of antibiotic discovery, ensembles of GNNs were paired with substructure-level attribution methods to identify molecular features associated with antibacterial activity. These learned rationales were then used to enrich for novel structural classes during prospective testing, including validation in mouse infection models [120]. More broadly, such approaches enable predictive models to function as data-driven sources of structure-activity hypotheses, facilitating fragment prioritization, liability mitigation, and scaffold innovation.

#### Generative ligand-based design and multi-objective optimization

The generative modeling frameworks based on variational autoencoders (VAEs) and adversarial learning established the feasibility of exploring underrepresented regions of chemical space using ligand-only training signals. These studies demonstrated that deep generative models could produce structurally novel molecules while retaining biologically relevant activity profiles, providing an initial proof-of-concept for data-driven molecular design beyond enumeration of known compounds [121, 122]. In particular, VAEs established the concept of continuous latent chemical spaces, where molecules are encoded as vectors and decoded into valid structures. This enables interpolation between chemotypes, smooth property optimization, and scaffold-level exploration along latent directions associated with potency or physicochemical endpoints [123].

Beyond small molecules, similar latent-space principles have been applied to peptide design. For example, a gated recurrent unit (GRU-based VAE combined with probabilistic latent-space sampling enabled the generation of target-specific peptide inhibitors to disrupt β-catenin/NEMO PPI [124] (Table 2).

Building on this foundation, generative AI models enable de novo molecular design by learning the underlying distribution of chemical space and sampling from it under explicit constraints. Unlike classical virtual screening, which ranks compounds from a fixed library, generative design creates new molecules. It can explore related analogs and guide designs toward multiple medicinal chemistry goals, such as potency, selectivity, and tractability [125, 126]. In practice, generative chemistry has proven most effective when treated as a hypothesis engine embedded within iterative design-make-test-analyze cycles, rather than as a one-shot molecule generator [126].

Among the most mature and widely adopted generative strategies are chemical language models that generate molecules like SMILES strings and can be fine-tuned on target-relevant ligand sets, enabling controlled exploration around known chemotypes while remaining compatible with medicinal chemistry constraints [127]. Large-scale transformer-based models such as MolGPT [128] exemplify this paradigm, demonstrating that autoregressive language models can learn chemically coherent distributions across millions of compounds and support conditional molecule generation. These models primarily advance architectural and representational capabilities, providing flexible generative backbones that can be coupled with downstream optimization and filtering pipelines. For example, a transformer-based chemical language model fine-tuned on receptor-specific ligand sets led to the discovery of new nanomolar A2A adenosine receptor ligands [129] (Table 2).

Recent advances in ligand-based generative design have extended conditional molecule generation beyond chemical and physicochemical constraints to incorporate functional genomic context. For example, G2D-Diff models generate hit-like small molecules directly from cancer genotypes and drug-response profiles, bypassing explicit target specification and transcriptomic input [130]. By learning genotype-response relationships within a diffusion framework, G2D-Diff produces chemically diverse compounds enriched for experimentally validated activity across multiple cancer contexts. This strategy illustrates how generative AI can operate at the interface of genomics and chemistry, enabling phenotype- and genotype-informed molecule design that complements target-centric and structure-based discovery paradigms, particularly in precision oncology settings where actionable targets may be ambiguous or context dependent. Another example of experimentally validated generative design is the discovery of dual ligands using a chemical language model (CLM) pretrained on ChEMBL and fine-tuned on selective ligands for four proteins: FXR, sEH, THRβ, and PPARδ. Separate models were trained for three target pairs (FXR/sEH, FXR/THRβ, and PPARδ/sEH) using pooled fine-tuning [131]. Twelve prioritized binders were synthesized and tested, showing activity on at least one target, and seven of them were confirmed as dual modulators, demonstrating the feasibility of CLM-based multi-target de novo design [131].

Complementary generative strategies frame lead optimization as a molecular translation task, in which a starting compound is converted into an improved analog under defined property constraints. Transformer-based SMILES-to-SMILES models extend beyond simple matched molecular pair transformations and enable more diverse, chemically meaningful modifications while optimizing for potency, selectivity, and physicochemical properties [132].

Reinforcement learning (RL) has been incorporated into generative models to support goal-directed molecular design. In this setting, the model proposes structural modifications and is rewarded based on predicted activity, while penalized for liabilities such as high lipophilicity or off-target risk. Early RL studies showed that models could generate analogs biased toward predefined property profiles while maintaining chemically reasonable structures [127]. More recent fragment-based and multiparameter RL approaches better reflect lead optimization practice by improving several properties simultaneously rather than optimizing a single metric. These strategies enable scaffold decoration and analog design workflows that retain potency while adjusting exposure, permeability, or clearance-related properties into acceptable ranges [133].

Generative models have also been applied to scaffold hopping, where key binding determinants are preserved while the central core is modified to improve intellectual property position, selectivity, safety, or ADMET. Historically, scaffold hopping relied on pharmacophore and topological search methods, with early algorithmic frameworks providing a foundation for systematic hopping strategies [134]. Deeplearning-based approaches such as DeepScaffold [135] extended this concept by explicitly learning scaffold transformations from data, enabling the generation of novel cores conditioned on reference ligands while preserving pharmacophoric features. More recent generative methods extend scaffold hopping by enforcing constraints such as similarity-to-parent, property windows, and three-dimensional shape or electrostatic consistency. This strategy is exemplified by DeepFMPO v3D [136], a fragment-based generative reinforcement-learning framework that incorporates explicit three-dimensional shape and electrostatic similarity through the ESP-Sim metric to guide multiparameter optimization. For instance, applied in a scaffold-hopping study on CDK2, this method identified bioisosteric core replacements that were not recoverable using two-dimensional similarity alone [136]. Likewise, DrugEx v2 applied Pareto-based multi-objective optimization with explicit control of off-target risk [137], and DrugEx v3 extended this strategy to scaffold-constrained design using a graph Transformer to grow and connect fragments from an input scaffold [138]. REINVENT 4 further integrates sequence- and transformer-based generators with reinforcement learning and curriculum learning to support de novo design, R-group replacement, linker design, scaffold hopping, and library design within a unified framework [139].

A key credibility test for generative ligand-based design is prospective experimental validation. For example, the deep generative framework GENTRL was used to identify potent DDR1 kinase inhibitors and opioid receptor ligands [140, 141]. Selected compounds were synthesized and showed confirmed biochemical and cellular activity, linking molecular generation with synthetic feasibility and experimental validation within a single workflow (Table 2). An entangled conditional adversarial autoencoder (ECAAE) framework enabled the semisupervised discovery of a selective JAK3 inhibitor with micromolar potency [142]. Similarly, hierarchical graph-based generative models have enabled the AI-driven discovery of nanomolar DYRK1A inhibitors [143] (Table 2). These studies demonstrate that ligand-based generative models can produce experimentally actionable hypotheses when objective functions, synthetic constraints, and experimental feedback are appropriately integrated.

However, generative models also have important practical constraints. Because they optimize predefined scoring functions, limitations in those metrics can produce misleading results, including chemically unstable or synthetically inaccessible structures, unless synthetic realism is explicitly incorporated [126]. For this reason, effective generative workflows combine molecular generation with predictive models for potency and tractability, explicit synthetic constraints, and experimental feedback. This integrated approach enables iterative refinement across successive design-make-test cycles [140].

## AI for difficult modalities

AI has significantly expanded the scope of small-molecule drug discovery into modalities that were historically considered difficult to drug, including protein-protein interactions, allosteric regulation, induced proximity therapeutics such as PROTACs, and molecular glues. These target classes are characterized by shallow or dynamic interfaces, context-dependent binding, and nonlinear structure-function relationships that challenge traditional medicinal chemistry strategies. By integrating structural biology, conformational dynamics, interaction energetics, and empirical success patterns, AI-driven approaches increasingly enable systematic exploration of these difficult modalities [144, 145].

### Protein-protein interaction inhibitors

PPIs typically involve large, relatively flat interfaces lacking the deep pockets favored by classical enzyme/drug discovery [146]. However, many PPIs are governed by localized “hot spots” that contribute disproportionately to binding free energy, providing tractable opportunities for small-molecule modulation. AI and machine-learning approaches are being increasingly applied to identify hot spots, infer interface features associated with ligandability, and prioritize chemotypes by learning from structural and experimental binding/functional data [147, 148].

A canonical example of PPI targeting is the MDM2/p53 interaction, where structure-guided discovery originally yielded the Nutlin class of inhibitors (e.g., Nutlin-3) that disrupt MDM2/p53 binding and reactivate p53 signaling [149]. While these early compounds predate modern deep learning, recent AI-enabled workflows have substantially expanded this paradigm by tightly integrating virtual screening with quantitative cellular PPI readouts. Notably, the NaviScreen platform, which combines Deep Site and Docking Pose (DSDP)-based AI-driven virtual screening with the NanaPPI technique for in situ measurement of endogenous PPIs, was applied to the p53/MDM2 system [150]. Screening ~ 3 million compounds yielded multiple cell-permeable inhibitors and identified a lead compound, AB-460 (Table 2), which disrupted endogenous p53/MDM2 PPI, increased p53 and p21 levels, suppressed tumor cell proliferation, and reduced tumor burden in vivo. Recent AI-enabled studies extended the PPI discovery framework beyond oncology. For instance, the integration of quantitative PPI assays, AlphaFold-Multimer structure prediction, and AI-guided ultra-large virtual screening enabled targeting of the NSP10-NSP16 interaction in SARS-CoV-2 [151]. This approach identified a conserved interface hot spot on NSP10 and yielded small-molecule inhibitors that disrupted complex formation, reduced methyltransferase activity, and suppressed viral replication in cells, illustrating how AI can systematically translate PPI hot spot identification into functional inhibition across diverse biological contexts.

### PROTACs

Recent analyses emphasize that PROTAC efficacy depends on the formation of a productive ternary complex between the target protein, the degrader, and an E3 ligase. Building on this framework, AI-driven approaches have begun to systematically address the intrinsic geometric and energetic complexity of degrader design through integrated structure-based modeling, generative design, and experimental validation [145].

For instance, AI-guided structural design frameworks have tackled PROTAC linker optimization by combining 3D deep neural network-based linker generation with explicit spatial constraints derived from known ternary complexes [152]. Superimposing alternative BRD4-ligand complexes onto the BRD4-AT7-VHL ternary structure enabled linker design under defined geometric constraints. Subsequent filtering by docking, buried solvent-accessible surface area, and binding free-energy estimates prioritized candidates for molecular dynamics and free-energy perturbation simulations. Using BRD4 as the target protein and VHL or cIAP1 as E3 ligases, this sequential strategy generated ternary complexes with predicted stability and binding energetics exceeding those of reference structures. Benchmarking against the established BRD4 degrader AT7 (DC_50_ ≈ 20.8 nM) further supported the approach for rational PROTAC optimization.

Another nanomolar BRD4 PROTAC was designed using a transformer-based generative framework coupled with reinforcement learning (PROTAC-RL), which optimized linker composition and pharmacokinetic properties prior to physics-based filtering and experimental validation [153]. Complementing such generative strategies, predictive deep graph models such as AiPROTAC have been developed to directly estimate PROTAC degradation capacity using multimodal graph encoders and cross-attention mechanisms, enabling AI-guided prioritization of androgen receptor degraders with experimental validation [154]. End-to-end generative AI strategies, such as Chemistry42 platform [65], further demonstrate how integration of molecular design with experimental validation can accelerate degrader development. For instance, Chemistry42 was applied to design a novel chemotype of PKMYT1 inhibitors by jointly optimizing structure-based interactions, kinase selectivity, and pharmacokinetic properties [155]. The optimized AI-generated inhibitor exhibited low-nanomolar potency and robust cellular suppression of CDK1 phosphorylation. Incorporation of this inhibitor into CRBN-recruiting PROTACs yielded degraders with sub-nanomolar PKMYT1 degradation potency (DC_50_ ~ 1 nM), enhanced pathway suppression relative to inhibition alone, high selectivity, and strong antitumor efficacy in xenograft models [155] (Table 2).

Beyond de novo design, AI has also enabled systematic repurposing and mechanistic insight through PROTAC-guided target identification. A multimodal deep learning framework integrating pathway-level prediction with transcriptomic modeling identified melatonin as a candidate modulator of aging- and Alzheimer’s disease-associated pathways from approved anti-insomnia drugs [156]. Subsequent PROTAC-enabled chemoproteomic profiling uncovered the histone acetyltransferase p300 as the direct molecular target, linking melatonin activity to SP1-dependent super-enhancer regulation of BMAL1. Extensive validation demonstrated suppression of neuroinflammation and senescence markers in vitro, improved cognition and pathology in Alzheimer’s disease mouse models, and an age-dependent decline of circulating melatonin in human cohorts [156].

### Molecular glues

Molecular glues stabilize or induce protein-protein interactions rather than inhibiting them, enabling degradation or functional rewiring of proteins that lack classical druggable pockets. Historically, most molecular glues, including thalidomide and its immunomodulatory derivatives (IMiDs) such as lenalidomide and pomalidomide, were discovered serendipitously, with design principles inferred only retrospectively. Recent AI-driven and structure-guided approaches are beginning to replace this trial-and-error paradigm with systematic, mechanism-informed discovery.

Recently, a data-driven structural bioinformatics framework, GlueFinder, was developed to systematically identify ligandable pockets adjacent to protein-protein interfaces across the Protein Data Bank [157]. By mining > 2600 human protein dimers, GlueFinder revealed that interface-adjacent pockets are widespread and can support gluemediated complex formation. The platform successfully recapitulated known glues, such as thalidomide binding at the CRBN-SALL4 interface with near-native accuracy, and predicted candidate molecular glues for therapeutically relevant targets, including EGFR, HER2, and KRAS, recruiting dozens to hundreds of distinct E3 ligases per target. These results suggest that glueable interfaces are not rare exceptions but a generalizable structural feature that can be computationally prioritized.

Complementing interface mining, recent work shows that AI-guided target prioritization and structure-based modeling can directly enable molecular glue discovery. An integrated AI framework combining QSAR, pathway analysis, docking, and MD identified bufalin as a molecular glue that stabilizes ERα-STUB1 interactions [158]. Structural and biochemical studies confirmed ERα binding (Kd ≈ 5.4 μM) and glue-induced complex stabilization, leading to potent ERα degradation at low-nanomolar concentrations, reversal of tamoxifen resistance, and efficacy in xenograft and patient-derived organoid models.

### Peptide Design

Peptides represent a distinct and increasingly important therapeutic modality, occupying an intermediate space between small molecules and biologics. Their ability to engage extended, shallow, or dynamic protein interfaces, combined with tunable specificity and modularity, makes them particularly attractive for targeting PPIs, membranes, and signaling pathways. Recent reviews have emphasized that artificial intelligence has fundamentally reshaped peptide discovery by enabling systematic navigation of the vast combinatorial sequence space and by integrating sequence, structure, and function into unified design frameworks [159, 160]. Early AI-driven peptide design efforts focused on sequence-based learning, where supervised deep learning models were trained on curated peptide datasets to predict activity, toxicity, stability, or spectrum of action. These approaches showed that neural networks out-performed rule-based and physicochemical filters in identifying functional peptides by learning higher-order sequence features linked to biological activity, thereby moving beyond simple motif reuse [159].

More recently, peptide design efforts have transitioned from predictive sequence modeling to generative approaches that learn the statistical and functional constraints of active peptides and propose de novo candidates. Diffusion-based and transformer-based frameworks have been particularly effective because they enable controlled exploration of peptide sequence space while preserving key physicochemical features. A latent diffusion model coupled to a transformer encoder was trained on curated antimicrobial peptide datasets to generate short, cationic, amphipathic peptides explicitly optimized for membrane-disruptive activity [161]. The resulting AI-designed peptides exhibited high sequence novelty while maintaining charge and hydrophobicity profiles associated with antimicrobial function. From a focused experimental set, multiple peptides showed low-micromolar to sub-micromolar antimicrobial activity against multidrug-resistant pathogens, and lead candidates demonstrated in vivo efficacy in mouse lung and skin infection models.

To address the limitations of sequence-only methods, recent models incorporate explicit structural information into peptide design. By integrating predicted peptide conformations and structural stability constraints into the generation process, recent models produced peptides optimized not only for activity but also for folding propensity, conformational stability, and productive target engagement [162]. Such strategies reduce the generation of misfolded or non-productive peptides and improve experimental hit rates by incorporating structural constraints into sequence design.

One of the major conceptual advances came with the introduction of RFdiffusion, which reframes peptide and mini-protein design as a conditional denoising process directly in three-dimensional space [163]. RFdiffusion enables de novo generation of peptide and protein binders conditioned on target geometry, explicitly encoding interface shape and hotspot complementarity. Experimentally, RFdiffusion-designed binders achieved high-affinity interactions with therapeutically relevant targets, including peptide scaffolds that mimic and substantially outperform native interaction motifs, demonstrating that AI can design functional peptide binders with atomic-level control over binding geometry. For example, this framework was used to design epitope-specific antibody domains, including VHHs and scFvs, targeting influenza haemagglutinin, *Clostridium difficile* toxin B, SARS-CoV-2 receptor-binding domain, and peptide-MHC complexes. Multiple designed binders were experimentally validated by surface plasmon resonance and cryo-EM, achieving nanomolar binding affinities and near-atomic agreement between predicted and measured structures [164]. These results demonstrate that diffusion-based structural generation can produce peptide and antibody binders with both high affinity and accurate binding geometry.

## ADMET prediction

Suboptimal absorption, distribution, metabolism, excretion, and toxicity (ADMET) remain a persistent bottleneck in drug development. AI-based ADMET models integrate chemical structures, physicochemical features, and biological data, ranging from in vitro assays to clinical pharmacokinetics, to predict liabilities earlier in the pipeline, enabling data-driven trade-offs between potency, efficacy, and developability [165].

Machine learning and deep learning frameworks, including graph neural networks (GNNs), message-passing neural networks (MPNNs), and transformer architectures, capture nonlinear relationships between molecular structure and ADMET properties. Recent studies have demonstrated improved predictive accuracy for endpoints such as passive permeability [166], P-glycoprotein (P-gp) efflux [167], CYP450 inhibition [168], and hepatotoxicity [169] using multimodal AI models trained on integrated chemical and biological datasets.

AI-driven ADMET prediction also supports multi-objective optimization, where efficacy, safety, and pharmacokinetics are jointly optimized during virtual screening. For example, integrated platforms such as ADMETlab [170] and ADMET-AI [171] employ multi-task learning to simultaneously predict absorption, clearance, and cardiotoxicity across dozens of endpoints, thereby guiding lead prioritization before in vivo testing. These platforms are increasingly benchmarked against drug-like chemical space, enabling rapid triage of compounds with unfavorable exposure or safety profiles. Moreover, the use of explainable AI (XAI) tools, such as integrated gradients and SHAP, has enabled mechanistic interpretation of model outputs, helping chemists identify molecular substructures linked to toxicity or metabolic instability rather than relying solely on black-box predictions [172].

The incorporation of large-scale clinical data and mechanistic knowledge has recently enabled more realistic prediction of drug-drug interactions (DDIs) and metabolism-related risks without relying solely on curated enzyme annotations. AI frameworks now combine chemical structure with pharmacological knowledge graphs, real-world evidence, and patient-level covariates to model DDIs prospectively across diverse populations [173]. Graph neural networks and multimodal learning approaches have been used to infer both pharmacokinetic and pharmacodynamic interaction mechanisms, improving detection of high-risk drug pairs beyond traditional rule-based systems [174]. In parallel, generative and systems-level models integrating transporter expression, enzyme competition, and exposure dynamics have demonstrated improved prediction of clinically relevant DDIs and clearance liabilities, including transporter-mediated effects and context-dependent metabolic inhibition [175].

## Retrosynthesis, synthetic feasibility, and reaction prediction

As AI-driven molecular design increasingly operates at the scale of ultra-large chemical spaces, coupling generation with synthetic feasibility has become essential for practical translation. Retrosynthesis and reaction-prediction models provide a critical bridge between in silico design and experimental execution by assessing whether proposed molecules can be synthesized using known chemistry and available building blocks.

AI-based retrosynthesis tools leverage reaction template libraries, graph-based representations, and sequence-to-sequence models to decompose target molecules into feasible precursors while accounting for reaction likelihood, step count, and reagent availability. Platforms such as ASKCOS [176] and AiZynthFinder [177] enable automated, multi-step retrosynthetic planning by integrating learned reaction rules with heuristic search, allowing evaluation of synthetic accessibility early in hit and lead discovery. Complementing these academic tools, IBM RXN for Chemistry [178] and Synthia [179] provide commercially deployed, AI-assisted retrosynthesis environments that combine transformer-based reaction prediction with curated reaction knowledge to propose experimentally grounded synthetic routes suitable for laboratory execution. To enable high-throughput feasibility assessment, RAscore [180] provides a machine-learning-based synthetic accessibility metric trained on retrosynthesis planner outcomes, rapidly estimating whether compounds are likely to be synthetically tractable without performing full route enumeration.

Complementing retrosynthesis, forward reaction prediction models learn to map reactants to products directly, enabling rapid evaluation of proposed transformations and supporting virtual reaction screening. Transformer-based architectures, exemplified by the Molecular Transformer [181] and Chemformer frameworks [182], achieve high accuracy in predicting reaction outcomes across diverse reaction classes, facilitating reaction feasibility assessment, yield estimation, and data-driven reaction optimization. Together, retrosynthesis and forward synthesis models enable closed-loop workflows in which molecule generation, synthetic route planning, and experimental validation are iteratively linked.

Importantly, integration of these tools with generative design platforms ensures that AI-proposed compounds are not only optimized for biological and physicochemical properties but are also grounded in realistic synthetic routes. As reaction databases expand and models increasingly incorporate cost, sustainability, and scalability considerations, AI-driven synthesis planning is poised to become a core component of end-to-end, experimentally actionable drug discovery pipelines.

## Limitations and future directions of AI-based target and drug discovery

Artificial intelligence has become a powerful integrative layer across both target discovery and small-molecule design, enabling the synthesis of biological complexity with chemical decision-making at unprecedented scale. As these approaches mature, their limitations are best viewed as natural frontiers arising from data incompleteness, biological context dependence, and experimental uncertainty, actively shaping the next phase of innovation rather than constituting barriers to adoption.

Despite rapid methodological progress, several practical limitations continue to shape the reliability and translational impact of AI-based discovery workflows. Training datasets are often biased toward well-studied targets and chemotypes, with a relative scarcity of high-quality negative data, which can skew model learning and inflate apparent performance. Distribution shift remains a persistent challenge, as models trained on cell lines or simplified assay systems do not always generalize to organoids, in vivo models, or patient samples. Label noise and assay variability, particularly in high-throughput screenings and phenotypic datasets, further complicate model training and can propagate uncertainty into downstream predictions. In addition, many deep learning models remain overconfident outside their domain of applicability, underscoring the importance of uncertainty estimation and calibrated confidence measures for decision-making. Finally, inconsistent benchmarking practices, dataset leakage, and limited prospective validation can limit reproducibility and complicate fair comparison across methods, underscoring the need for more consistent benchmarking protocols and experimentally grounded evaluation criteria.

In target discovery, AI excels at integrating diverse biological signals, such as genomics, transcriptomics, perturbation data, and network context, to prioritize functionally relevant targets beyond simple association or differential expression. However, challenges remain in separating true disease drivers from correlated but non-functional signals, particularly in heterogeneous diseases and sparse clinical settings. Advances in multimodal and perturbation-aware AI are helping to mitigate these limitations by elevating functionally supported targets. As single-cell, spatial, and longitudinal datasets expand, AI models are increasingly positioned to resolve cell-type-specific vulnerabilities and dynamic disease programs with greater precision.

In small-molecule discovery, generative and predictive models now support rapid exploration of chemical space, multiparameter optimization, and rational design of compounds and combinations. Key limitations, including synthetic accessibility, assay translatability, and uncertainty in predicted affinities, are being addressed through tighter integration of synthetic feasibility, retrosynthetic planning, and experimental feedback. Closed-loop workflows that combine molecular design, biological testing, and model retraining are emerging as a unifying paradigm, enabling AI systems to learn directly from experimental outcomes and iteratively refine both chemical hypotheses and biological understanding.

Across both domains, interpretability and mechanistic insight are becoming defining features of next-generation AI systems. Rather than functioning as opaque predictors, modern models increasingly provide structured explanations, linking molecular features to biological pathways, network effects, or phenotypic outcomes, thereby supporting hypothesis-driven experimentation and medicinal chemistry decision-making. This shift is particularly important for translational settings, where mechanistic clarity facilitates target validation, combination rationale, and regulatory confidence.

Looking forward, the convergence of foundation models, multimodal learning, and physics-informed approaches is expected to further unify target discovery and molecular design into coherent, end-to-end discovery platforms. These systems will increasingly operate across scales, from molecular interactions to cellular networks and clinical phenotypes, enabling context-aware discovery strategies that adapt to disease biology rather than relying on fixed heuristics. Importantly, human expertise remains central in this ecosystem: domain knowledge guides model objectives, interprets outputs, and defines meaningful experimental tests that drive learning forward.

In summary, the future of AI in drug discovery may lie less in automation alone and more in deeper integration, linking targets to chemistry, predictions to experiments, and data-driven inference to mechanistic understanding. As datasets, models, and workflows continue to evolve together, AI is likely to become an increasingly important tool for identifying actionable biological targets and supporting their translation into effective, well-designed therapeutics.

## Conclusion

Artificial intelligence is increasingly embedded across early drug discovery, providing an integrative framework that connects biological target rationale with practical chemical decision-making. Importantly, AI-based ADMET prediction, DDI risk modeling, and synthetic feasibility assessments are being incorporated earlier in design cycles, enabling developability-aware prioritization rather than latestage triage. In target discovery, network- and multimodal AI approaches strengthen the prioritization of actionable drivers by combining genetic and molecular context with functional evidence from perturbation and dependency resources. In parallel, AI-enabled structure-based and ligand-based methods accelerate hit identification and multiparameter optimization, particularly when coupled with AI-guided retrosynthetic planning, feasibility scoring, and iterative experimental feedback. These advances are extending medicinal chemistry into challenging modalities, including PPIs, induced proximity therapeutics, molecular glues, and peptides, where traditional heuristics often fall short. Despite rapid progress, translating AI predictions into experimentally validated discoveries depends on data quality, context-relevant models, reliable estimation of uncertainty, and mechanistic interpretability. The greatest impact is likely to arise from integrated discovery workflows that combine AI-driven design with iterative experimental cycles, enabling models to learn from experimental outcomes while remaining guided by medicinal chemistry expertise. Overall, AI is emerging as a powerful tool for improving prioritization, expanding chemical exploration, and increasing efficiency in generating high-quality, synthetically tractable, and developable therapeutic hypotheses.

## Figures and Tables

**Table 1:** Representative artificial intelligence platforms and computational frameworks for target discovery and molecular design

Name	Description	Ref
*Network- and Systems-Based Target Discovery & Interactome Modeling*		
NDEx (Network Data Exchange)	Platform for sharing, versioning, and programmatic access to biological networks as computable objects with explicit provenance and reproducibility support	[12]
OncoPPi	Cancer-focused protein-protein interaction network enabling identification of non-enzymatic and regulatory cancer targets	[14, 15]
AVERON	AI platform for identifying mutation-induced neomorphic protein-protein interactions (neoPPIs) and linking them to oncogenic programs	[18]
diffPALM	Sequence-based protein-protein interaction inference using masked protein language modeling on paired protein sequences	[22]
InteracTor	Protein language model-based framework for proteome-scale interaction prediction with explainable interaction features	[23]
SPIDER*	Graph attention-based framework integrating global interactomes with condition-specific molecular context to infer cell-type-specific protein-protein interaction networks	[24]
Random-walk / diffusion methods	Network propagation approaches that spread disease-associated signals across interaction networks to prioritize functionally related genes and pathways	[17]
Graph Neural Networks (GNNs)	Graph-based AI models integrating network topology and node attributes for dependency discovery, link prediction, and module detection	[20, 21]
Discriminative Network Embedding (DNE)	Contrastive self-supervised learning of protein-protein interaction topology enabling functional module discovery without node-level annotations	[25]
*Perturbation-Aware Functional Target Discovery*		
CM4AI (Cell Maps for Artificial Intelligence)	Large-scale perturbation, proteomics, interaction mapping, and phenotyping resource supporting AI-ready functional target discovery	[51]
PDGrapher	Neural network framework identifying network-effective intervention sets that optimally shift diseased transcriptional states	[52]
Geneformer	Single-cell foundation model enabling in silico gene perturbation and identification of dosage-sensitive disease regulators	[53]
PINNACLE	Contextual AI framework integrating single-cell transcriptomics with protein-protein interaction networks for cell-type-specific target prioritization	[54]
DepMap	Genome-scale CRISPR dependency resource for identifying cancer-specific genetic vulnerabilities	[56]
TCGA-DEPMAP	Machine-learning framework projecting cell-line dependency data onto patient tumors to identify clinically relevant dependencies	[57]
*Structure Prediction and Complex Modeling*		
AlphaFold3	Diffusion-based framework for predicting protein-protein, protein-ligand, and nucleic-acid complexes	[88]
Boltz-2	Foundation model integrating structure prediction and affinity estimation for protein-ligand and protein-protein interactions	[103, 104]
RoseTTAFold All-Atom (RFAA)	Unified all-atom deep learning model for end-to-end prediction of protein-ligand and multicomponent complexes	[99]
Chai-1	Diffusion-based co-folding model for protein-ligand complex prediction evaluated in prospective crystallographic benchmarks	[106]
*AI-Assisted Docking and Pose Estimation*		
GNINA	Convolutional neural network-based pose rescoring and refinement integrated into classical docking pipelines	[95, 96],
Deep Docking	Surrogate docking strategy using deep regression models to enable ultralarge virtual screening	[97]
RosettaVS	AI-accelerated docking framework combining learned scoring with physics-based structural sampling	[6]
*Affinity Prediction and Energetics Modeling*		
KDEEP	Three-dimensional convolutional neural network for protein-ligand binding affinity prediction	[100]
Pafnucy	Deep learning framework for structure-based binding affinity estimation	[101]
PotentialNet	Graph neural network for protein-ligand affinity and molecular property prediction	[102]
SiteAF3	Conditional diffusion framework for site-specific protein-ligand and protein-biomolecule complex prediction	[107]
*Benchmarking and Evaluation Frameworks*		
MoleculeNet	Standardized benchmarking platform and dataset collection for molecular machine learning, enabling reproducible evaluation of graph-based and representation-learning models across QSAR, biophysics, and physiological tasks	[113]
CASP Benchmarks	Community-wide blind evaluations defining performance limits of structure and complex prediction models	[93]
*Generative Ligand-Based Design*		
MG-BERT	SMILES-based chemical language model for molecular property prediction	[111]
FG-BERT	Functional group-aware molecular language model aligned with medicinal chemistry abstractions	[112]
MolGPT	Transformer-based molecular language model for de novo molecule generation	[128]
G2D-Diff	Genotype-to-drug diffusion model generating small molecules from cancer genotypes and drug-response profiles	[130]
DeepScaffold	Scaffold-based generative framework for de novo molecular design	[135]
DeepFMPO v3D	3D shape- and electrostatics-constrained generative reinforcement learning for multiparameter optimization	[136]
DrugEx (v2, v3)	Scaffold-aware multi-objective reinforcement learning framework for molecular generation	[137, 138]
REINVENT 4	Production-grade platform supporting de novo design, scaffold hopping, and linker generation	[139]
GENTRL	Deep generative framework with prospective experimental validation	[141]
*Chemical Perturbation and Closed-Loop Discovery*		
Connectivity Map (CMap / L1000)	Large-scale perturbational transcriptomics resource linking genes, pathways, and small molecules	[61]
DrugReflector	Transcriptomics-driven active learning framework for identifying modulators of disease phenotypes	[62]
CSG^2^A	Transfer learning framework integrating perturbation, drug response, and clinical data	[63]
PandaOmics	Multimodal AI platform integrating omics, clinical data, and knowledge graphs for target prioritization	[64]
Chemistry42	Generative chemistry platform integrating active learning with experimental feedback	[65]
*Druggability and Binding-Site Prediction*		
DrugProtAI	Sequence-based machine learning framework for proteome-wide druggability prediction	[67]
DrugnomeAI	Ensemble learning framework for target-level druggability prediction	[68]
XGB-DrugPred	Gradient-boosting model for druggable protein classification	[69]
P2Rank	Machine learning-based ligand-binding site detection	[72]
DeepSite	Three-dimensional convolutional neural network for binding pocket prediction	[73]
DeepPocket	Deep learning-based pocket detection and rescoring	[74]
DRLiPS	Machine learning framework for predicting druggable RNA-small-molecule binding pockets	[75]
SPIDER*	A stacked ensemble-learning model that integrates diverse sequence-derived features and classifiers to improve robustness and interpretability of protein druggability prediction.	[71]
*Chemical Space Databases and Enumeration Resources*		
GDB-17	Explicitly enumerated database of 166.4 billion chemically stable small molecules with up to 17 heavy atoms, providing a lower bound for feasible chemical space	[78]
PubChem	Public repository of synthesized compounds with biological assay annotations, enabling large-scale ligand-based modeling	[81]
ChEMBL	Curated bioactivity database supporting QSAR, multitask learning, and benchmark model training	[82]
Enamine REAL / WuXi GalaXi	Make-on-demand, reaction-based virtual libraries enabling screening of tens of billions of synthetically accessible compounds	[84]
*AI for PPI Inhibitor Discovery*		
NaviScreen	AI-guided ultra-large virtual screening platform integrated with in situ cellular PPI quantification (NanaPPI)	[149]
NanaPPI	Live-cell assay platform for direct measurement of endogenous protein-protein interactions	[149]
**Molecular Glue Discovery**		
GlueFinder	Structural bioinformatics platform identifying ligandable pockets adjacent to protein-protein interfaces for molecular glue discovery	[157]
*Peptide and Mini-Protein Design*		
RFdiffusion	Conditional diffusion framework for de novo peptide and mini-protein binder design in 3D space	[163]
Diffusion-based peptide generators	Generative peptide design frameworks producing experimentally validated antimicrobial and signaling peptides	[161]
Structure-aware peptide generators	AI models integrating folding stability and conformational constraints into peptide generation	[162]
*ADMET and DDI Prediction Platforms*		
ADMETlab	Multi-task AI platform for predicting absorption, clearance, toxicity, and cardiotoxicity	[170]
ADMET-AI	Integrated deep-learning framework for joint ADMET endpoint prediction	[171]
DDI-GNN frameworks	Graph-based and multimodal AI systems for predicting pharmacokinetic and pharmacodynamic drug-drug interactions	[173, 174]
*Retrosynthesis & Synthetic Feasibility Tools*		
ASKCOS	Open-source AI synthesis planning suite supporting multi-step route evaluation	[176]
AiZynthFinder	Neural network-guided retrosynthesis planner that breaks down targets into purchasable precursors with route suggestions	[177]
IBM RXN for Chemistry	Cloud AI platform for retrosynthesis and reaction outcome prediction using transformer models	[178]
Synthia	Commercial retrosynthesis planner integrating curated rules and AI search for robust synthetic routes	[179]
RAscore	ML-based synthetic accessibility score derived from retrosynthesis planning output for fast feasibility estimation	[180]
Molecular Transformer	Transformer-based sequence-to-sequence model for forward reaction prediction, enabling accurate reactant-to-product mapping, reaction feasibility assessment, and yield-aware reaction outcome prediction across diverse reaction classes	[181]
Chemformer	Unified transformer framework for reaction prediction, retrosynthesis, and molecular optimization, supporting bidirectional modeling of chemical reactions and integration into closed-loop synthesis planning workflows	[182]

Note that the SPIDER software reported in Refs. [24] and [71] corresponds to two diff erent tools.

**Table 2:** Examples of AI-discovered and experimentally validated small molecules

Compound	Target	Reported Activity	AI/ML Method	Ref
**Enzyme inhibitors**				
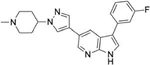	DYRK1A	IC_50_ = 41 nM	Hierarchical Graph Generation (HGG) model	[143]
3-(3-fluorophenyl)-5-(1-(1-methylpiperidin-4-yl)-1H-pyrazol-4-yl)-1H-pyrrolo[2,3-b] pyridine. (Compound 1)				
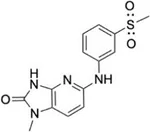	JAK3	IC_50_ = 6.73 μM	Entangled Conditional Adversarial Autoencoder	[142]
1-methyl-5-((3-(methylsulfonyl)phenyl) amino)-1,3-dihydro-2H-imidazo[4,5-b] pyridin-2-one				
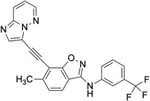	DDR1	IC_50_ = 10 nM	GENTRL	[141]
7-(imidazo[1,2-b]pyridazin-3-ylethynyl)-6-methyl-N-(3-(trifluoromethyl)phenyl) benzo[d]isoxazol-3-amine. (Compound 1)				
**GPCR ligands**				
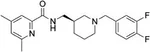	κ1 opioid receptor	pKi = 5.19	Modified GENTRL	[140]
(S)-N-((1-(3,4-difluorobenzyl)piperidin-3-yl) methyl)-4,6-dimethylpicolinamide. (FJRL-14)				
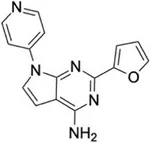	A_2A_ receptor	pKi = 7.5; pIC50 = 7.7	Chemical Language Models (CLM)	[129]
2-(furan-2-yl)-7-(pyridin-4-yl)-7H-pyrrolo[2,3-d]pyrimidin-4-amine. (Compound 9)				
**Molecular glues**				
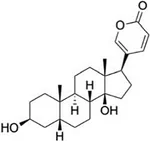	ERα/STUB1	Kd = 5.4 μM	Graph Neural Network (GNN)	[158]
5-((3S,5 R,8 R,9S,10S,13 R,14S,17 R)-3,14-dihydroxy-10,13-dimethylhexadecahydro-1H-cyclopenta[a]phenanthren-17-yl)-2H-pyran-2-one (Bufalin)				
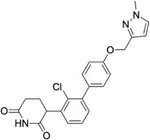	CRL4^CRBN^/VAV1	Low nanomolar potency	Protein Data Bank and AlphaFold2	[87]
3-(2-chloro-4’-((1-methyl-1H-pyrazol-3-yl) methoxy)-[1,1’-biphenyl]-3-yl)piperidine-2,6-dione. (Compound 6)				
**PPI inhibitors**				
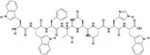	β-catenin/NEMO	IC_50_ = 0.01 μM	Gated Recurrent Unit VAE/Metropolis-Hastings (MH) latent space sampling.	[124]
Trp-Trp-Phe-Thr-Asp-Asp-His-Trp (CAL-2)				
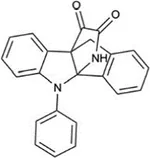	p53/MDM2	Low micromolar potency	Deep Site and Docking Pose (DSDP)-based virtual screening	[150]
5-phenyl-5H,10H-4b,9b-(epiminoethano) indeno[1,2-b]indole-11,12-dione. (AB-460)				
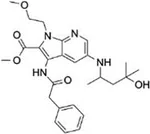	PD-1/PD-L1	IC_50_ = 17.53 μM	ML-QSAR	[119]
methyl 5-((4-hydroxy-4-methylpentan-2-yl) amino)-1-(2-methoxyethyl)-3-(2-phenylacetamido)-1H-pyrrolo[2,3-b] pyridine-2-carboxylate. (PDA13)				
**PROTACs**				
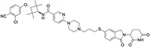	Androgen Receptors	DC_50_ ≤ 100 nM	AiPROTAC	[154]
N-((1r,3r)-3-(3-chloro-4-cyanophenoxy)-2,2,4,4-tetramethylcyclobutyl)-6-(4-(3-((2-(2,6-dioxopiperidin-3-yl)-1-oxoisoindolin-5-yl)thio)propyl)piperazin-1-yl)nicotinamide. (GT19)				
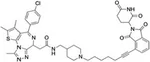	BRD4	IC_50_ = 116 nM	PROTAC-RL	[153]
2-(4-(4-chlorophenyl)-2,3,9-trimethyl-6H-thieno[3,2-f][1,2,4]triazolo[4,3-a][1,4] diazepin-6-yl)-N-((1-(7-(2-(2,6-dioxopiperidin-3-yl)-1,3-dioxoisoindolin-4-yl)hept-6-yn-1-yl)piperidin-4-yl)methyl) acetamide (Compound 1)				
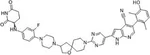	PKMYT1	DC_50_ = 0.7 nM	Chemistry42	[155]
2-(2-(3-(4-(4-(((R)-2,6-dioxopiperidin-3-yl) amino)-2-fluorophenyl)piperazin-1-yl)-1-oxa-8-azaspiro[4.5]decan-8-yl)pyrimidin-5-yl)-5-(3-hydroxy-2,6-dimethylphenyl)-1H-pyrrolo[2,3-b]pyridine-4-carbonitrile (D16-M1P2)				

## Data Availability

No datasets were generated or analysed during the current study.

## Box 1 Representative AI-based methods for target discovery

### Random Walk methods.

These approaches explore protein-protein interaction networks by repeatedly “walking” between interacting proteins. Proteins that are frequently encountered together are inferred to participate in related biological pathways, helping identify new disease-relevant targets by network proximity. **The model learns** the network reachability and global topology. Simulating movement across the edges of a graph, it captures the “statistical distance” between proteins.

### Graph Neural Networks (GNNs).

GNNs learn protein representations by systematically combining information from each protein and its interaction partners. This allows direct modeling of how local interaction neighborhoods influence protein function, disease relevance, or drug response. **The model learns** relational context and local neighborhood dynamics. It encodes how a protein’s identity is shaped by the specific neighbors within a functional cluster.

### Large Protein Language Models (PLMs).

PLMs are trained on millions of protein sequences and learn patterns related to protein structure, function, and evolution directly from sequence data. The resulting embeddings can be used alone or combined with PPI networks to improve target and pathway analysis. **The model learns** the “biological grammar” of sequences. By analyzing millions of amino acid sequences, it learns the evolutionary rules that govern whether a sequence folds correctly and performs a specific function.

### Discriminative Network Embedding (DNE).

DNE methods learn protein representations by explicitly separating closely interacting proteins from distant or unrelated ones in the network. This improves the ability to distinguish functionally relevant targets from background network noise. The model learns an optimal contrast and signal-to-noise separation. It focuses on finding the boundaries that define a functional group, learning to ignore biological “background.”

## Box 2 Representative AI-based methods for small-molecule discovery

### Convolutional Neural Networks (CNNs).

CNNs learn spatial patterns in protein-ligand complexes by analyzing local atomic interaction environments within binding pockets. By applying convolutional filters across three-dimensional structural representations, these models capture geometric and physicochemical complementarity between ligands and targets. They are commonly used for docking pose refinement and binding affinity prediction. The model learns spatial interaction signatures that link molecular structure to potency.

### Graph Neural Networks (GNNs).

GNNs represent small molecules as atom-bond graphs and iteratively update atomic features through message passing along chemical bonds. This allows the model to learn structure-activity relationships directly from molecular topology without predefined descriptors. GNNs are widely applied in QSAR, ADMET prediction, and multiparameter optimization. The model learns how local chemical environments shape global molecular properties.

### Transformer-Based Molecular Language Models.

Transformers treat molecular strings (e.g., SMILES) as sequences and use attention mechanisms to capture long-range dependencies between atoms and substructures. These models can learn chemically meaningful embeddings and support both property prediction and molecule generation. Autoregressive variants generate molecules token-by-token, while masked variants learn contextual representations through self-supervision. The model learns the “syntax” and statistical grammar of chemical structure.

### Variational Autoencoders (VAEs).

VAEs encode molecules into continuous latent spaces from which new molecules can be generated by interpolation and optimization. By learning probabilistic representations, VAEs enable smooth navigation of chemical space and property-guided exploration. They are commonly used in de novo molecular design and scaffold innovation. The model learns a structured latent representation of chemical space.

### Diffusion Models.

Diffusion models generate molecules by gradually denoising random noise into chemically valid structures. In structure-aware settings, they can be conditioned on binding pocket geometry or physicochemical constraints. These models are particularly effective for 3D molecule and complex generation. The model learns the generative trajectory that transforms noise into plausible chemical structures.

### Reinforcement Learning (RL) for Molecular Optimization.

RL frameworks optimize molecule generation using reward functions defined by predicted potency, selectivity, or ADMET properties. A policy network proposes molecular edits and is iteratively improved based on objective-driven feedback. RL is often layered onto generative models to enable goal-directed design. The model learns how to modify molecules to maximize predefined multi-objective criteria.

### Ensemble Tree-Based Models (Random Forest, Gradient Boosting).

These models aggregate multiple decision trees to improve predictive robustness in structured chemical descriptor space. They remain widely used in classical QSAR and ADMET modeling, particularly in low-data regimes. The model learns nonlinear relationships between molecular features and biological outcomes with strong generalization properties.

### Sequence-to-Sequence Reaction Models.

Sequence-to-sequence neural architectures translate reactant strings into product predictions or propose retrosynthetic steps. Using attention-based mechanisms, they learn chemical transformation rules directly from reaction data. These models support reaction prediction and synthetic route planning. The model learns mappings between chemical inputs and outputs at the reaction level.
